# 

**Published:** 1889-01-12

**Authors:** 


					J*
PERMANENTLY ENLARGED TO 32 PAGES.
ICE ONE PENNY. w?   /T
tfo. 120, Vol. v.-
-January 32th, 1889.
Per Annum, 6s. da., post free. u
TUTontVilir Porta ftrl ? nnct. fpAA 71(f.
_ ?, muiiuiiy coils. uu. j pooi. nee, iau.
L?e*l?tered at the General Pout Office f*fh_;
as a Newspaper.] Ditto per Ann. 7b. 6d? post free.

				

